# The Mitochondria is the Source of Hepatic Amyloid Precursor Protein and Peripheral Amyloid Beta: Implications of Alcohol‐induced Liver Steatosis in Alzheimer's Disease

**DOI:** 10.1002/alz70855_097269

**Published:** 2025-12-23

**Authors:** Josephine Chu, Ross A. Steinberg, Brian Carson, Devaraj V. Chandrashekar, Rachita K. Sumbria, Derick Han

**Affiliations:** ^1^ Keck Graduate Institute, Claremont, CA, USA; ^2^ School of Pharmacy, Chapman University, Irvine, CA, USA

## Abstract

**Background:**

Alcohol‐induced liver injury occurs in the pericentral region of the liver and can induce mitochondrial remodeling and exacerbate Alzheimer's Disease (AD) progression. Subpopulations of mitochondria: general mitochondria (GM), peridroplet mitochondria (PDM) and endoplasmic reticulum(ER)‐bound mitochondria (ERM) maintain cellular homeostasis via energy synthesis, lipid homeostasis, and regulation of intracellular calcium. Hepatic amyloid precursor protein (APP) is a source of peripheral amyloid beta (aB) and affects AD pathology in the brain. Understanding the localization and expression of hepatic APP in mitochondrial subpopulations are critical to understanding aB metabolism and may play a role in identifying a potential mechanism for metabolic dysfunction. Here, site‐specific expression of hepatic APP and aB processing proteins are measured in the subpopulations of the mitochondria of ethanol‐fed AD mice to identify the regions where aB metabolism occurs.

**Method:**

Double transgenic (APP/PS1) AD mice were intra‐gastric fed with ethanol or control diet for 5 weeks (*n* = 7‐11/group). Liver tissue was harvested for digital spatial profiling (DSP) analysis to measure pathogenic AD biomarkers (APP, PSEN1, BACE1) in the periportal and perivenous regions using sequence‐specific fluorescent probes. Mitochondrial fractions were isolated using differential centrifugation and homogenized for protein measurement using western blotting.

**Result:**

Hepatic APP expression increased 2‐fold and PSEN1 increased over 5‐fold in the mitochondria compared to the cytosol of both ethanol‐fed and non‐ethanol fed AD mice (*p*≤0.05). APP expression increased in the GM and ERM but not in the PDM of ethanol‐fed mice compared to control (*p*≤0.05). APP transcript increased in the pericentral region of ethanol‐fed mice (*p*≤0.05) and both APP and aB showed an increased trend at the protein level (*p* = 0.06 and *p* = 0.07, respectively).

**Conclusion:**

Differential expression of APP across mitochondrial subpopulations suggests that hepatic mitochondria may be a source of peripheral aB and affect specific mitochondrial processes in the GM and ERM. Increased expression of APP in the pericentral region of AD mice correlates to the same site affected in alcohol‐induced liver damage and may assist in identifying potential pathways involved in AD progression due to alcohol‐induced liver impairment. Future studies modulating hepatic APP *in vivo* will provide future insights on the contribution of hepatic aB on AD pathogenesis.

